# The Mutation Glu151Asp in the B-Component of the *Bacillus cereus* Non-Hemolytic Enterotoxin (Nhe) Leads to a Diverging Reactivity in Antibody-Based Detection Systems

**DOI:** 10.3390/toxins7114655

**Published:** 2015-11-09

**Authors:** Andrea Didier, Nadja Jeßberger, Victoria Krey, Richard Dietrich, Siegfried Scherer, Erwin Märtlbauer

**Affiliations:** 1 Department of Veterinary Science, Faculty of Veterinary Medicine, Ludwig-Maximilians Universität München, 85763 Oberschleißheim, Germany; E-Mails: n.jessberger@mh.vetmed.uni-muenchen.de (N.J.); r.dietrich@mh.vetmed.uni-muenchen.de (R.D.); e.maertlbauer@mh.vetmed.uni-muenchen.de (E.M.); 2 Lehrstuhl für Mikrobielle Ökologie, Zentralinstitut für Ernährungs-und Lebensmittelforschung, Wissenschaftszentrum Weihenstephan, Technische Universität München, 85354 Freising, Germany; E-Mails: vk@borreliosecentrum.de (V.K.); siegfried.scherer@wzw.tum.de (S.S.)

**Keywords:** *Bacillus cereus*, non-hemolytic enterotoxin B, Duopath^®^, point mutation

## Abstract

The ability of *Bacillus cereus* to cause foodborne toxicoinfections leads to increasing concerns regarding consumer protection. For the diarrhea-associated enterotoxins, the assessment of the non-hemolytic enterotoxin B (NheB) titer determined by a sandwich enzyme immunoassay (EIA) correlates best with *in vitro* cytotoxicity. In general, the regulation of enterotoxin expression of *B. cereus* is a coordinately-regulated process influenced by environmental, and probably also by host factors. As long as these factors are not completely understood, the currently-applied diagnostic procedures are based on indirect approaches to assess the potential virulence of an isolate. To date, sandwich EIA results serve as a surrogate marker to categorize isolates as either potentially low or highly toxic. Here, we report on a single amino acid exchange in the NheB sequence leading to an underestimation of the cytotoxic potential in a limited number of strains. During the screening of a large panel of *B. cereus* isolates, six showed uncommon features with low sandwich EIA titers despite high cytotoxicity. Sequence analysis revealed the point-mutation ^Glu^151^Asp^ in the potential binding region of the capture antibody. Application of this antibody also results in low titers in an indirect EIA format and shows variable detection intensities in Western-immunoblots. A commercially-available assay based on a lateral flow device detects all strains correctly as NheB producers in a qualitative manner. In conclusion, isolates showing low NheB titers should additionally be assayed in an indirect EIA or for their *in vitro* cytotoxicity to ensure a correct classification as either low or highly toxic.

## 1. Introduction

*B. cereus sensu stricto*, is one of eight closely related species forming the *B. cereus sensu lato* group, additionally comprising *B. thuringiensis*, *B. weihenstephanensis*, *B. mycoides*, *B. pseudomycoides*, *B. cytotoxicus*, *B. toyonensis* and, finally, the potentially bio-terroristic agent *B. anthracis*. Aside from its ability to cause food spoilage, the ubiquitous soil organism *B. cereus* is progressively involved in food poisoning [1]. Within the European Union the number of outbreaks increased by 122% in 2011 [2]. Among the frequently-detected *B. cereus* isolates, a broad variety of strains exists, ranging from highly toxic to atoxic ones [3,4]. Determining the toxic potential of an uncharacterized isolate is, therefore, an urgent demand with regard to food safety concerns. The widely used culture-based methods on selective media, like PEMBA or MYP, are based on the quantification of typical colonies resulting in the diagnosis of presumptive *B. cereus* (International Organization for Standardization - ISO7932). This definition indicates that not only *B. cereus sensu stricto* but also other members of the *B. cereus sensu lato* group might be present. The procedure reflects well that enterotoxins are also produced by other *B. cereus* group members. Especially *B. thuringiensis* strains have been reported to express the non-hemolytic enterotoxin (Nhe) as well as hemolysinBL (Hbl) [5]. On the other hand the ISO method does not include an approach to address toxicity and is, therefore, not suited to categorize different isolates according to their toxic potential towards humans. Such a classification will become necessary in the future [6], as ubiquitous *B. cereus* cannot completely be eradicated from the food chain.

The gastrointestinal illnesses associated with *B. cereus*, and resulting in diarrheal symptoms, are mainly caused by two enterotoxin complexes. The Nhe toxin [7], consisting of the components NheA, NheB, and NheC, is present in almost all isolates [8] whereas the Hbl complex [9] is found in approximately 50% of the isolates. To characterize a *B. cereus* strain with regard to the presence of the enterotoxins, a broad panel of methods is available. Classical [10,11] or real-time [12] PCR-based approaches serve to determine the genetic background of toxin profiles. Due to the improvements of primer pairs applied, the former problem of false-negative results could be overcome [13]

To address the question of toxin expression on the protein level, commercial, or in-house test systems based on immunochemical detection methods must be used. The commercially-available assays are: (i) a reversed passive latex agglutination assay (Oxoid™ RPLA, Wesel, Germany), (ii) the *Bacillus* diarrheal enterotoxin visual immunoassay (BDE VIA™ Tecra, St. Paul, MN, USA), and (iii) the Duopath^®^ Cereus Enterotoxins test (Merck, Darmstadt, Germany). The latter assay is based on gold-labeled monoclonal antibodies in a lateral flow device.

As a proof of functionality the *in vitro* cytotoxicity of a strain can finally be determined in cell-based systems [14]. For ethical reasons, these assays have replaced *in vivo* cytotoxicity tests, e.g., the rabbit ileal loop test [15] or the vascular permeability assay [16]. Namely, Vero cells are used in these assays, but applicability has also been shown for several other cell lines [4]. Especially, the NheB titers detected in a sandwich EIA correlate well with the cytotoxic effects on Vero cells [4,15] and can, therefore, indicate the potential virulence towards humans. For the second enterotoxin complex (Hbl), EIA titers of the L1 and the B-Component correlate best with the *in vitro* cytotoxicity [4]. Recently, it was reported that the regulation of enterotoxin production is highly complex and not yet fully understood [17]. As long as no further virulence markers are identified the NheB titers are still best-suited to categorize an isolate. According to the correlation of NheB titers and *in vitro* toxicity levels *B. cereus* isolates are currently sorted as having high toxicity or low toxicity. This artificial categorization is based on the amount of enterotoxin (detected on the protein level) produced under defined laboratory conditions optimized for toxin expression, and the subsequent determination of *in vitro* cytotoxicity. However, recently a small proportion of strains (approx. 2%) was observed, which does not fit these categories. Those strains show low titers in the in-house sandwich EIA despite a medium-to-strong cytotoxic potential towards Vero cells. As a corollary, such an isolate will be underestimated during risk-assessment or in an outbreak investigation scenario, in the event that only the NheB protein level, and not the cytotoxic potential, is assayed. Based on our long-standing experience on antibody-based detection systems and the large number of *B. cereus* strains characterized we hypothesized that at least the epitope of one of the antibodies applied is altered in the “suspicious” strains. A systematic approach comprising *nheB* sequencing, EIAs, Western blot, and cytotoxicity assays now enables us to understand this surprising phenomenon.

## 2. Results

### 2.1. B. Cereus Strains with Uncommon Reactivity Pattern

During the typing of more than 200 strains from the collections in Munich and Freising we became aware of six isolates with a strong discrepancy between sandwich EIA titers and cytotoxicity levels on Vero cells. All strains were solely Nhe-producers as tested by EIAs. The two parameters (EIA titers and cytotoxicity level) are known to correlate well, thus enabling the discrimination of strains with high and low toxicity. This discrepancy prompted us to elucidate the background of this rare and uncommon phenomenon. The Nhe-high producing strain MHI 241 (NVH0075/95; Nhe-reference strain) and one further strain (MHI3178) known to express Nhe at very low levels, were additionally included as controls. Furthermore, the present study comprised three strains, which showed an intermediate reactivity in the NheB-specific sandwich-EIA during a former project but have neither been tested in the indirect EIAs nor in the cytotoxicity assays so far. The background information on the origin of the strains, as well as the most important sequencing results introduced later in detail, are summarized in Table 1.

**Table 1:** Strains investigated in this study. The last column shows the results of sequencing. WB: Western blot.

Strain Number	Origin	Type	AA or Mutation at Position 151
MHI 241	stew with vegetables	high toxicity	Glu
MHI 3178	infant food	low toxicity	Glu
MHI 1440	unknown	^151^mutant	Glu › Asp
MHI 1541	unknown	^151^mutant	Glu › Asp
MHI 1668	infant food	^151^mutant	Glu › Asp
MHI 2970	milk powder	^151^mutant	Glu › Asp
MHI 3173	human faeces	^151^mutant	Glu › Asp
MHI 3225	coffee cream	^151^mutant	Glu › Asp
MHI 1430	unknown	intermediate performance in EIA and WB	Glu
MHI 1444	unknown	intermediate performance in EIA and WB	Glu
MMI 1758	unknown	intermediate performance in EIA and WB	Glu

Table 2 summarizes the performance of the Vero cytotoxicity assays, the results of the different in-house EIAs applied, and Duopath^®^ test.

**Table 2:** Cytotoxicity and toxin titers. All strains have been tested by the Vero, as well as CaCo-2 cytotoxicity assay, an in-house sandwich EIA, and two in-house indirect EIAs. Titers indicated are means and SD of triplicates. The strains reacted positively in the Duopath^®^ test; Duopath^®^ tests were performed twice.

Strain	Cytotoxicity titer (Vero cells)	Cytotoxicity titer (CaCo-2 cells)	Sandwich EIA titer	Indirect EIA 1E11 titer	Indirect EIA 2B11 titer	Duopath^®^
MHI 241	1381 (±8.0)	358 (±46.2)	27072 (±2841.9)	2669 (±128.7)	118 (±0.7)	+
MHI 3178	16 (±4.2)	4 (±0.3)	192 (±7.8)	113 (±9.2)	27 (±2.8)	+
MHI 1440	218 (±20.5)	202 (±39.2)	5.4 (±0.2)	583 (±33.9)	2.6 (±1.3)	+
MHI 1541	533 (±46.0)	198 (±22.4)	19 (±1.4)	775 (±76.4)	6.7 (±1.1)	+
MHI 1668	628 (±36.8)	157 (±26.6)	47.5 (±0.7)	2330 (±753.1)	47.5 (±0.7)	+
MHI 2970	755 (±4.2)	345 (±80.2)	41.5 (±2.1)	1204 (±125.2)	11 (±1.3)	+
MHI 3173	1210 (±253.1)	271 (±46.3)	38.5 (±2.7)	1682 (±14.1)	5.7 (±2.0)	+
MHI 3225	1274 (±247.5)	200 (±10.2)	36.5 (±0.7)	1692 (±14.1)	6.9 (±1.3)	+
MHI 1430	679 (±89.0)	280 (±15.4)	467 (±26.9)	2383 (±501.3)	51 (±9.9)	+
MHI 1444	740 (±162.3)	508 (±60.9)	445 (±85.6)	2591 (±413.0)	47 (±2.8)	+
MHI 1758	1053 (±215.0)	699 (±109.0)	961 (±56.6)	2755 (±166.2)	52 (±4.2)	*

+ positive reaction; * not analyzed.

For the classically high- and low-producing strains MHI 241 and MHI 3178, a consistent reaction pattern was observed showing either high or low titers in the different EIA setups, which correspond well to the high or low cytotoxicity data, respectively.

In contrast, six strains (MHI 1440, 1541, 1668, 2979, 3173, and 3225) revealed medium-to-high cytotoxicity on Vero cells levels despite a weak performance in the sandwich EIA, as well as in the indirect EIA based on mAb 2B11. On the other hand, results obtained in an indirect EIA based on mAb 1E11 showed medium to high titers. The three further strains (MHI 1430, 1444 and 1758) revealed an intermediate performance in the sandwich EIA as well as in the mAb 2B11-based indirect assay. The corresponding titers obtained in the mAb 1E11-based indirect EIA, as well as the levels of cytotoxicity, were high. Furthermore, all supernatants were assayed on CaCo-2 cells revealing lower titers than on Vero cells, but an overall similar reaction pattern. Statistical analysis by t-test showed that the very low titers received from the six strains in the sandwich and the mAb 2B11-indirect EIA differ significantly (*p* < 0.001) from those of the three intermediate performing strains. On the other hand, cytotoxicity levels did not show significant differences (*p* = 0.72). These results gave a first hint that the poor sandwich- and 2B11-based indirect EIA performance, despite high cytotoxicity, might be caused by impaired reactivity of mAb 2B11.

Therefore, all strains under study were subjected to sequencing of the *nheB* gene in order to search for mutations in the putative antibody epitopes.

### 2.2. Sequencing of the NheB Gene Unravels a Point Mutation at Position 151

**Figure 1:**
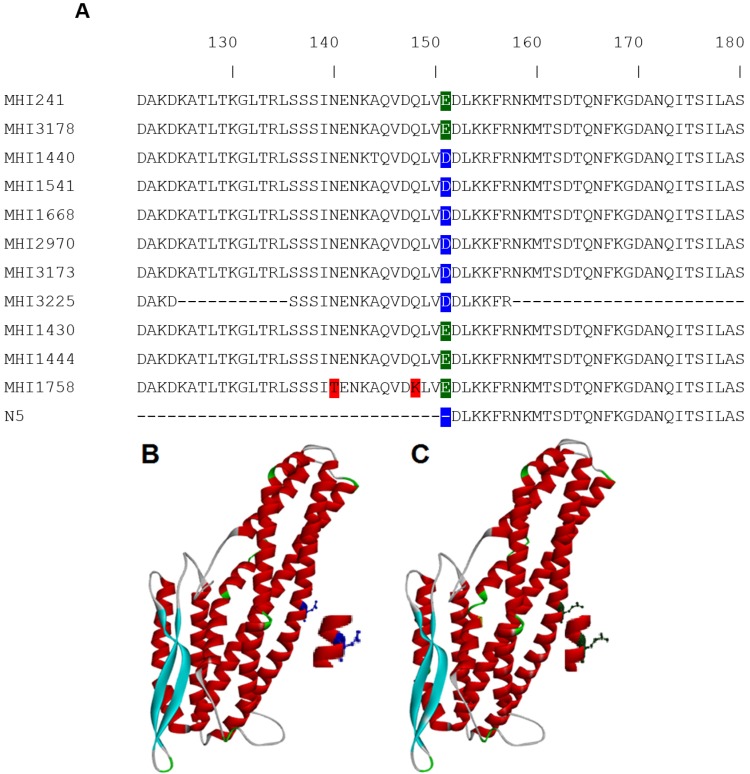
(**A**) Partial NheB sequences comprising amino acid 121–180. The complete Clustal Omega alignment file is given as Figure S1. Although several primers were applied, sequencing of MHI 3225 was only partially possible for unknown reasons. The most prominent difference is the point mutation at position ^E^151^D^ compared to the reference strain MHI 241. This mutation is not present in a formerly-published rNheB fragment, which always tested negative in EIA and Western blot; (**B**) position of the aspartic acid residue in the structural model of NheB; and (**C**) the glutamic acid residue is protuding more and slightly rotated in the model.

Results were subsequently translated to the amino acid sequence and compared by the Clustal Omega program [18] (Figure 1A). The most prominent difference consistently found in all six strains showing a uncommonly weak 2B11 reactivity was a single amino acid (aa) exchange at position 151 (^E^151^D^). In Figure 1B,C this position is highlighted in the structural model of NheB. The recently-resolved structure of NheA (PDB-ID: 4K1P) [19] served as a template to create a NheB model on the SWISS-MODEL server (Swiss Institute of Bioinformatics and University of Basel, Basel, Switzerland).

The three strains with an intermediate performance (MHI 1430, MHI 1444, and MHI 1758) did not bear this mutation, although they have amino acid exchanges between position 230 and 250 (see: complete alignment in the Supplementary file (Figure S1)).

### 2.3. Performance of the Mutant Strains in a Lateral Flow Device

**Figure 2:**
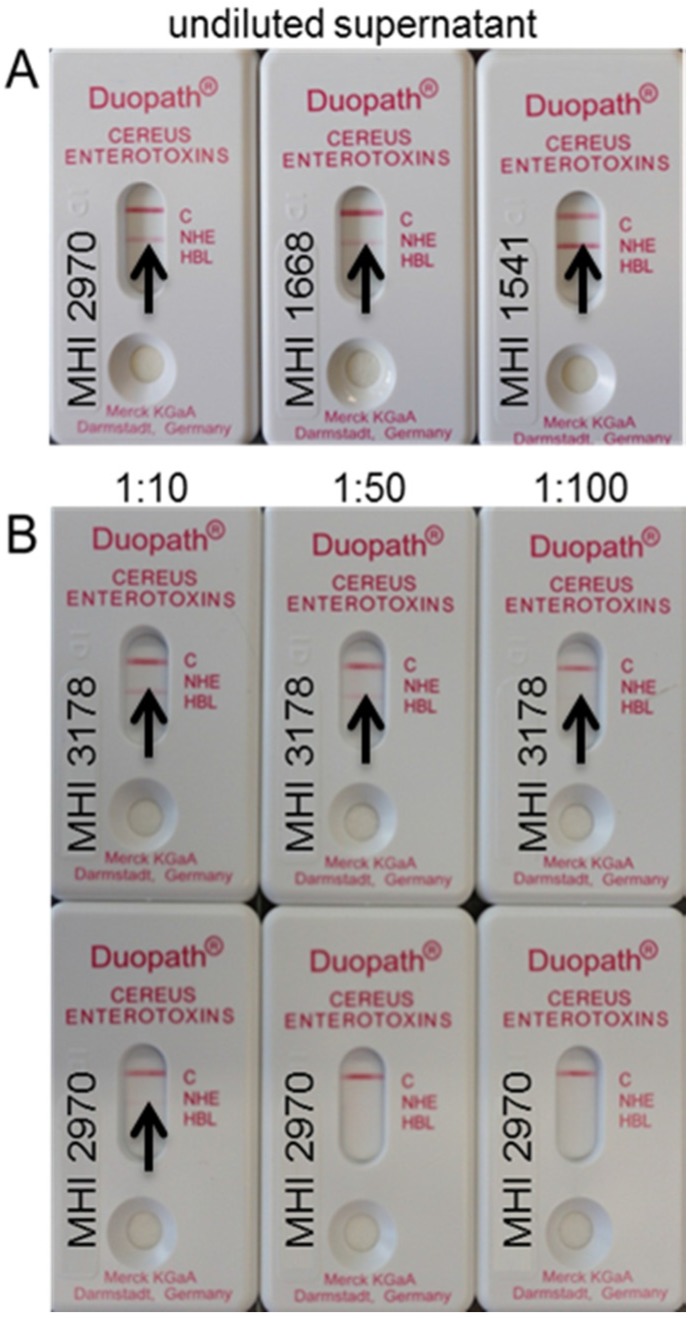
(**A**) Examples of the Duopath^®^ Assay results for the mutant strains MHI 2970, MHI 1668, and MHI 1541. Undiluted supernatants were added to the lateral flow device. All strains showed a positive reaction for Nhe; (**B**) dilution series of a typical low-producer (MHI 3178) compared to one of the mutant strains (MHI 2970). A positive reaction is visible up to a dilution of 1:100 for the low-producer but only up to 1:10 for the mutant strain. The arrows point to the NheB band; the pink C on the device shows control band.

In order to assay the consequences of the point mutation in a further test system, the Duopath^®^ assay was performed. Interestingly, all strains under study reacted positively in the Duopath® test (Table 1, examples depicted in Figure 2A). Only when a dilution series of the supernatants was applied, the signal of the mutant strains faded out earlier than that of the low-producer MHI 3178 (comparatively depicted for MHI 2970 in Figure 2B). These results are interesting since the mAbs included in the Duopath^®^ test are the 1E11 and 2B11.

### 2.4. Western Blot Reactivity of the Mutants

**Figure 3:**
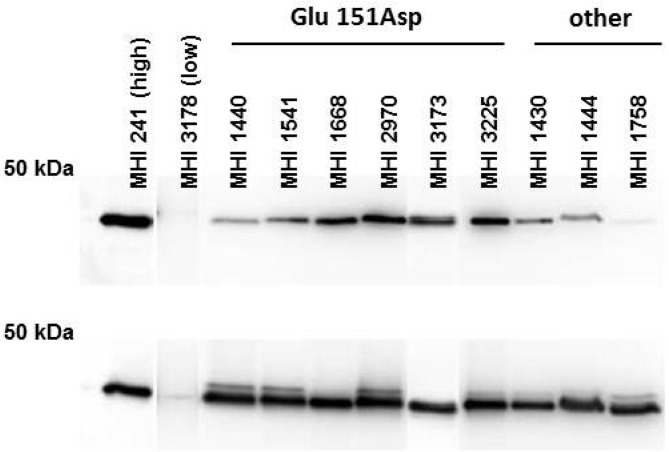
Western blot results of the membranes probed with mAB 2B11 (upper panel) and 1E11 (lower panel). Except for the low-producer, for which an adjustment of the NheB amounts was impossible, all strains reacted similarly with mAb 1E11. Loaded amounts were adjusted to contain similar NheB levels as calculated based on the indirect EIA results. Under denaturing conditions mAb 2B11 is capable of detecting NheB in all supernatants, with varying intensities. Mean signal for the probed membrane with mAb 1E11 was 219 RLU (±18.5), and 145 RLU (±70.2) for mAb 2B11, thus indicating a lower reactivity, as well as a higher degree of variability for this antibody.

To further assay the antibody performance under denaturing conditions, Western immunoblots were carried out on samples adjusted to equal amounts of NheB according to the results of the indirect EIA (mAb 1E11). One membrane was probed with mAb 1E11 to control the loading of equal protein amounts. A second membrane was probed with mAb 2B11. Results depicted in Figure 3 show that, except for the low NheB producer MHI, 3178 mAb 1E11 reacts equally well with all strains under study. Interestingly, NheB is detectable in all strains to varying degrees irrespective of the weak performance in the 2B11-based EIAs wherein—under non-denaturing conditions—the protein is targeted by the antibody.

## 3. Discussion and Conclusions

Due to its increasing contribution to food poisoning outbreaks in recent years, *B. cereus* has become a hygienic and technological problem in the food industry. The total elimination of this ubiquitous microorganism from the food chain cannot be guaranteed. All *B. cereus* isolates described so far—including the probiotic strain *B. cereus var. toyoi* [20]—possess the genetic background for at least one of the three-component enterotoxin-complexes. Together with the increasing knowledge on the complex regulatory network of toxin expression and the diverse lifestyle of *B. cereus* isolates [21], several approaches aim to elucidate which parameters lead to disease in humans. For risk assessment of uncharacterized isolates the herein proposed artificial categorization of “highly“ and “low” toxic strains based on defined and optimized laboratory conditions is a promising approach to address the protein level. Assaying the genetic background by PCR as a rapid and high-throughput method tends to be advantageous for epidemiological tests during an outbreak scenario. Thus, both methods have their special qualification.

On the one hand, the number of ingested bacterial cells or spores seems to be important to progress from a coincidental contamination to gastrointestinal disease [22]. On the other hand, recent developments aim to characterize a strain in order to find predictive markers for its cytotoxic potential. These efforts include the search for additional virulence factors, e.g., HlyII, InhA1, NprA [23], or sphingomyelinase [24], as well as studies on the survival of bacterial cells and productivity of toxins under conditions mimicking the gastrointestinal tract [25]. For the Nhe-toxin complex the highest predictive value on the protein level with regard to the potential cytotoxicity is given by the amount of the NheB component [4]. As all of the strains introduced in the present study are solely Nhe-producers the contribution of Hbl to the total cytotoxicity could be ruled out.

A recent publication described the differential contribution of the Nhe and Hbl-toxin complexes to the total cytotoxicity on different cell lines. On Vero cells, Nhe accounts for approx. 60% of the total toxicity, whereas Hbl accounts for the remaining 40%. On CaCo-2 both enterotoxin complexes contributed equally [4]. This prompted us to consider Vero cells as the cell line of choice because of its sensitivity and because of the fact that all strains presented herein are solely Nhe producers.

The low reactivity of six strains in an in-house-sandwich EIA, despite high cytotoxicity, is an uncommon phenomenon and it was reasonable to assume that the binding of at least one of the mAbs applied was impaired. The epitope which is targeted by mAb 1E11 (NSLLQNVDSISPNDLVFIKE) has been mapped in a former study [26] is not mutated in any of the strains. In the same publication the potential 2B11 epitope could only be assigned to a broad range of amino acids (aa 122–151) and conformation dependency could not be excluded completely. By unraveling the point mutation ^E^151^D^ in the six mutant strains the assumption on the epitope location made earlier was confirmed and further supported by the fact that mAb 2B11 was never reactive towards a recombinantly-expressed NheB fragment (N5—described in detail also in [27]) deleted by 151 aa from the N-terminus. Interestingly, mAb 2B11 is able to detect NheB of the mutant strains, to varying degrees, under the denaturing conditions of a Western blot (Figure 3). This feature is in good accordance with the results published earlier. It further indicates that the impaired antibody binding due to the point mutation can partially be overcome by SDS and Reducing agent^®^ (Biorad, Hercules, CA, USA) causing protein denaturation under Western immunoblotting conditions. Thus, the reactivity of mAb 2B11, aside from the primary amino acid sequence, also seems to be conformation dependent.

In Figure 1B,C, the critical amino acid exchange is highlighted in the structural model of NheB. Although glutamic acid and aspartic acid are both characterized by an additional carboxyl-group, and the total charge is not affected, the more protruding glutamic acid seems to be important for effective antibody binding. To the best of our knowledge, impaired antibody binding as a consequence of this mutation has not been described in the literature. However, the occurrence of altered enzyme or protein function, as well as viral tropism due to respective single amino acid exchange, has been reported [27,28,29].

The fact that the mutant strains are still detectable in the Duopath^®^ assay can be explained by the technical setup of the lateral flow device. Herein, the undiluted supernatant is applied and washing steps are not included. Thus, the weakly-bound mAb 2B11 is not prone to being washed off. The less-stable binding of mAb 2B11 due to a mutated NheB becomes obvious when diluted supernatants of mutants were applied in the Duopath^®^ test. For comparison, diluted supernatants of the low-producing/low toxicicty MHI 3178 were also tested. The NheB-specific band of the low-producer is still detectable at higher dilutions (Figure 2B upper panel), although the amount of protein is lower, whereas the signal of a mutant strain already faded out (Figure 2B lower panel). It is suggested that this is the case because the lower amount of NheB present in MHI 3178 will be compensated by a stronger binding of 2B11.

The uncommon reaction pattern of six NheB mutant strains in an in-house sandwich EIA point out a problem that could be faced when a categorization of a *B. cereus* isolate of high toxicity or low toxicity under conditions optimized for toxin expression is intended, but only a fast and easy to accomplish sandwich EIA is performed. Strains showing low sandwich EIA titers might be sorted to be weakly toxic by relying only on a general correlation between NheB titers and *in vitro* toxicity. This phenomenon was observed in a minor percentage of strains (app. 2%) investigated in this study, which is in agreement with an analysis of 142 genome sequences of *Bacillus cereus sensu lato* [30], revealing the presence of this mutation in three strains.

As long as no other markers for the categorization in high and low toxin producers are available, a solution of the problem could be as follows: uncharacterized isolates being “suspicious” due to a poor performance in the sandwich EIA should, additionally, be assayed by an indirect EIA based on mAb 1E11 and/or by *in vitro* cytotoxicity assays. An important result of the present study is that mutant strains will not go completely undetected since they react positively in the Duopath^®^ test. Thus, they will be correctly typed as Nhe producers, though only qualitatively.

## 4. Experimental Section

### 4.1. Bacterial Strains and Culture

Prior to inclusion in this study all strains were tested to express the Nhe-toxin complex components but not Hbl, as determined by EIA. For toxin production, a 1% inoculum of overnight cultures was subcultured at 32 °C in casein-hydrolysate yeast broth supplemented with 1% glucose. After six hours, bacterial supernatants were harvested by centrifugation at 4000 g at 4 °C for 20 min, supplemented with 1 mM EDTA and passed through a 0.2 µm sterile filter (Millipore, Darmstadt, Germany). Aliquots of the cell-free supernatants were stored at −20 °C.

### 4.2. DNA Preparation and Sequencing of NheB

The cell pellets of 200 µL overnight cultures were subjected to genomic DNA extraction (Blood and Tissue kit, Qiagen, Hilden, Germany) according to the manufacturer’s protocol optimized for Gram-positive bacteria. Sequencing of the *nheB* genes was performed by GATC Biotech GmbH (Konstanz, Germany). Translation of nucleotide sequences and Clustal Omega alignment was carried out via the web service of the EMBL-EBI [18].

### 4.3. Indirect and Sandwich EIA

Maxisorb microtitre plates (Nunc, Wiesbaden, Germany) were coated overnight at room temperature with serial dilutions of cell-free *B. cereus* supernatants. After 45 min of blocking in 3% (*w*/*v*) casein-phosphate buffered saline (PBS) primary antibodies (1µg/mL of 1E11; 2 µg/mL of 2B11) were incubated for 1 h at room temperature. After four washing steps with 150 mM NaCl 0.025% Tween 20, rabbit-anti mouse-HRP conjugate (DAKO, Hamburg, Germany) at a dilution of 1:2000 was applied for 1 h. Plates were washed five times followed by 3,3′,5,5′-tetramethylbenzidine (TMB) (Sigma, St. Louis, MO, USA) incubation for 20 min. After adding 100 µL 1 M H_2_SO_4_, OD was determined in a TECAN (Männedorf, Switzerland) EIA reader at 450 nm with a 620 nm reference filter. For the sandwich EIA, plates were coated with mAb 2B11 (5 µg/mL) overnight. After blocking, serially-diluted samples (dilution buffer 0.5% Tween-PBS) were added for 1 h at room temperature. Three washing steps in 150 mM NaCl 0.025% Tween 20 were followed by incubation with mAb 1E11-HRP (1:2000) for another hour. Signal development and recording was done as described for the indirect EIA.

### 4.4. Duopath^®^ Cereus Enterotoxins Test

Supernatants of all strains under study were twice-subjected to Duopath^®^ Assay (Merck, Darmstadt, Germany) according to the manufacturer’s instructions. Additionally, the dilution series of the supernatants were assayed in this system. The latter procedure is not recommended by the manufacturer but it was necessary to elucidate different binding properties of mAb 2B11 as described in the results section.

### 4.5. Western Blot

SDS-PAGE was carried out twice on 12% Criterion Gel (Biorad, Hercules, CA, USA). Subsequent semidry blotting was followed by an incubation of the membrane in 3% (*w*/*v*) casein-phosphate buffered saline (PBS) for 1 h at room temperature. After this blocking step either mAb 1E11(1µg/mL) or mAb 2B11 (2 µg/mL) was applied on the membranes for 1 h. A rabbit anti-mouse-HRP secondary antibody (1:2000 (DAKO, Hamburg, Germany)) was added for 1 h after three times washing in 0.05% (*v*/*v*) PBS-Tween. Membranes were washed three times in PBS-Tween and twice in PBS. After addition of SuperSignal femto chemiluminescence substrate (Pierce, Schwerte, Germany), signals were recorded using a Kodak Image Station (Eastman Kodak, Rochester, NY, USA). Background corrected signal intensities were determined as relative light units (RLU) by the means of TotalLab^TM^ software (Version 2003.03, TotalLab, Newcastle upon Tyne, UK, 2003). Means and SD, as well as mean difference comparing results obtained with mAb 2B11 *versus* 1E11 were calculated.

### 4.6. Cytotoxicity Assay

Vero cells used in the cytotoxicity assays were maintained in MEM medium (Biochrom AG, Berlin, Germany) supplemented with 1% FCS (Sigma, St. Louis, MO, USA), 1% Na-Pyruvat (Biochrom AG, Berlin, Germany), and Pen/Strep (Biochrom AG, Berlin, Germany). CaCo-2 cells were cultured in RPMI medium supplemented with 10% FCS. After trypsinizing and adjusting the cell number to 15,000/well for Vero cells and 20,000/well for CaCo-2 cells, respectively, cell-free *B. cereus* supernatants were serially diluted on the microtiter plates and allowed to rest 24 h on the cells. Metabolic activity of the cells was determined after addition of 10 µL/well WST-1 reagent (Roche, Mannheim, Germany) for 2 h. Optical density at 450 nm with 620 nm reference filter was recorded on a TECAN (Männedorf, Switzerland) reader.

### 4.7. Statistics

*T*-tests were performed on SigmaPlot version 8.0 (SPSS Inc., Chicago, IL, USA, 2002).

## Supplementary Materials

Supplementary materials can be accessed at: http://www.mdpi.com/2072-6651/7/11/4655/s1.
