# Supplementary material for: The Mutation Glu151Asp in the B-Component of the Bacillus cereus Non-Hemolytic Enterotoxin (Nhe) Leads to a Diverging Reactivity in Antibody-Based Detection Systems
Source: Toxins (Basel). 2015 Nov 9;7(11):4655–67. doi: 10.3390/toxins7114655 (PMC4663526; doi:10.3390/toxins7114655)
Supplement: Supplementary file 1 [file toxins-07-04655-s001.pdf]

Supplementary Information

```
                  10        20        30        40        50        60
                   |         |         |         |         |         |
MHI241   AESTVKQAPVHAVAKAYNDYEEYSLGPEGLKDAMERTGSNALVMDLYALTIIKQGNVNFG
MHI3178  AESTVKQAPVHAVAKAYNDYEEYSLGPEGLKDAMERTGSNALVMDLYALTIIKQGNVNFG
MHI1440  AESTVKQAPVHAAAKAYNDYEEYSLGPEGLKDAMERTGSNALVMDLYALTIIKQGNVNFG
MHI1541  AESTVKQAPVHAVAKAYNDYEEYSLGPEGLKDAMERTGSNALVMDLYALTIIKQGSVNFG
MHI1668  AESTVKQAPVHAAAKAYNDYEEYSLGPEGLKDAMERTGSNALVMDLYALTIIKQGNVNFG
MHI2970  AESTVKQAPVHAAAKAYNDYEEYSLGPEGLKDAMERTGSNALVMDLYALTIIKQGNVNFG
MHI3173  AESTVKQAPVHAAAKAYNDYEEYSLGPEGLKDAMERTGSNALVMDLYALTIIKQGNVNFG
MHI3225  AESTVNQAPVHAVTKASNDDQEYSLRTDRLNHAMEIKSSDVLVLDLYTL-----------
MHI1430  AESTVKQAPVHAVAKAYNDYEEYSLGPEGLKDAMERTGSNALVMDLYALTIIKQGNVNFG
MHI1444  AESTVKQAPVHAVAKAYNDYEEYSLGPEGLKDAMERTGSNALVMDLYALTIIKQGNVNFG
MHI1758  AESTVKQAPVHAVAKAYNDYDEYSLGPEGLKDAMERTGSNALVMDLYALTIIKQGNVNFG
N5       ------------------------------------------------------------

                  70        80        90       100       110       120
                   |         |         |         |         |         |
MHI241   NVSSVDAALKGKVIQHQDTARGNAKQWLDVLKPQLISTNQNIINYNTKFQNYYDTLVAAV
MHI3178  NVSSVDAALKGKVIQHQDTARGNAKQWLDVLKPQLISTNQNIINYNTKFQNYYDTLVAAV
MHI1440  NVSSVDAALKGKVIQHQDTARGNAKQWLDVLKPQLISTNQNIINYNTKFQNYYDTLVAAV
MHI1541  NVSSVDAALKGKVIQHQDTARGNAKQWLDVLKPQLISTNQNIINYNTKFQNYYDTLVAAV
MHI1668  NVSSVDAALKGKVIQHQDTARGNAKQWLDVLKPQLISTNQNIINYNTKFQNYYDTLVAAV
MHI2970  NVSSVDAALKGKVIQHQDTARGNAKQWLDVLKPQLISTNQNIINYNTKFQNYYDTLVAAV
MHI3173  NVSSVDAALKGKVIQHQDTARGNAKQWLDVLKPQLISTNQNIINYNTKFQNYYDTLVAAV
MHI3225  ----------------------NAKQWLDVLKPQLISTNQNIINYNTKFQNYYDTLVAAV
MHI1430  NVSSVDAALKGKVIQHQDTARGNAKQWLDVLKPQLISTNQNIINYNTKFQNYYDTLVAAV
MHI1444  NVSSVDAALKGKVIQHQDTARGNAKQWLDVLKPQLISTNQNIINYNTKFQNYYDTLVAAV
MHI1758  NVTSVDTSLKGKVIQHQDTARGNAQQWLDVLKPQLISTNQNIINYNTKFQNYYDTLVAAV
N5       ------------------------------------------------------------

                 130       140       150       160       170       180
                   |         |         |         |         |         |
MHI241   DAKDKATLTKGLTRLSSSINENKAQVDQLVEDLKKFRNKMTSDTQNFKGDANQITSILAS
MHI3178  DAKDKATLTKGLTRLSSSINENKAQVDQLVEDLKKFRNKMTSDTQNFKGDANQITSILAS
MHI1440  DAKDKATLTKGLTRLSSSINENKTQVDQLVDDLKRFRNKMTSDTQNFKGDANQITSILAS
MHI1541  DAKDKATLTKGLTRLSSSINENKAQVDQLVDDLKKFRNKMTSDTQNFKGDANQITSILAS
MHI1668  DAKDKATLTKGLTRLSSSINENKAQVDQLVDDLKKFRNKMTSDTQNFKGDANQITSILAS
MHI2970  DAKDKATLTKGLTRLSSSINENKAQVDQLVDDLKKFRNKMTSDTQNFKGDANQITSILAS
MHI3173  DAKDKATLTKGLTRLSSSINENKAQVDQLVDDLKKFRNKMTSDTQNFKGDANQITSILAS
MHI3225  DAKD--------TRISSSINENKAQVDQLVDDLKKFR-----------------------
MHI1430  DAKDKATLTKGLTRLSSSINENKAQVDQLVEDLKKFRNKMTSDTQNFKGDANQITSILAS
MHI1444  DAKDKATLTKGLTRLSSSINENKAQVDQLVEDLKKFRNKMTSDTQNFKGDANQITSILAS
MHI1758  DAKDKATLTKGLTRLSSSITENKAQVDKLVEDLKKFRNKMTSDTQNFKGDANQITSILAS
N5       -------------------------------DLKKFRNKMTSDTQNFKGDANQITSILAS

                 190       200       210       220       230       240
                   |         |         |         |         |         |
MHI241   QDAGIPLLQNQITTYNEAISKYNAIIIGSSVATALGPIAIIGGAVVIATGAGTPLGVALI
MHI3178  QDAGIPLLQNQITTYNEAISKYNAIIIGSSVATALGPIAIIGGAVVIATGAGTPLGVALI
MHI1440  QDAGIPLLQNQITTYNEAISKYNAIIIGSSVATALGPIAIIGGAVVIATGAGTPLGVALI
MHI1541  QDAGIPLLQNQITTYNEAISKYNAIIIGSSVATALGPIAIIGGAVVIATGAGTPLGVALI
MHI1668  QDAGIPLLQNQITTYNEAISKYNAIIIGSSVATALGPIAIIGGAVVIATGAGTPLGVALI
MHI2970  QDAGIPLLQNQITTYNEAISKYNAIIIGSSVATALGPIAIIGGAVVIATGAGTPLGVALI
MHI3173  QDAGIPLLQNQITTYNEAISKYNAIIIGSSVATALGPIAIIGGAVVIATGAGTPLGVALI
MHI3225  -------------------------------------IAIIGGAVVIATGAGTPLGVALI
MHI1430  QDAGIPLLQNQITTYNEAISKYNAIIIGSSVATALGPIAIIGGAVVIATGAGTPLGVALI
MHI1444  QDAGIPLLQNQITTYNEAISKYNAIIIGSSVATALGPIAIIGGAVVIATGAGTPLGVALI
MHI1758  QDAGIPLLQNQITTYNEAISKYNAIIIGSSVATALGPIAIIGGAVVIATGAGTPLGIALI
N5       QDAGIPLLQNQITTYNEAISKYNAIIIGSSVATALGPIAIIGGAVVIATGAGTPLGVALI
```

Figure S1. *Cont.*

```
                      250       260       270       280       290       300
                        |         |         |         |         |         |
MHI241       AGGAAAVGGGTAGIVLAKKELDNAQAEIQKITGQVTTAQLEVAGLTNIKTQTEYLTNTID
MHI3178      AGGAAAVGGGTAGIVLAKKELDNAQAEIQKITGQVTTAQLEVAGLTNIKTQTEYLTNTID
MHI1440      AGGAAAVGGGTAGIVLAKKELDNAQAEIQKITGQVTTAQLEVAGLTNIKTQTEYLTNTID
MHI1541      AGGAAAVGGGTAGIVLAKKELDNAQAEIQKITGQVTTAQLEVAGLTNIKTQTEYLTNTID
MHI1668      AGGAAAVGGGTAGIVLAKKELDNAQAEIQKITGQVTTAQLEVAGLTNIKTQTEYLTNTID
MHI2970      AGGTAAVGGGTAGIVLAKKELDNAQAEIQKITGQVTTAQLEVAGLTNIKTQTEYLTNTID
MHI3173      AGGAAAVGGGTAGIVLAKKELDNAQAEIQKITGQVTTAQLEVAGLTNIKTQTEYLTNTID
MHI3225      AGGAAAVGGGTAGIVLAKKELDNAQAEIQKITGQVTTAQLEVAGLTNIKTQTEYLTNTID
MHI1430      AGGTAAVGGGTAGIVLAKKELDNAQAEIQKITGQVTTAQLEVAGLTNIKTQTEYLTNTID
MHI1444      AGGTAAVGGGTAGIVLAKKELDNAQAEIQKITGQVTTAQLEVAGLTNIKTQTEYLTNTID
MHI1758      AGGTAAVGGGTAGIVLAKKELDNAQAEIQKITGQVTTAQLEVAGLTNIKTQTEYLTNTID
N5xxxx11     AGGAAAVGGGTAGIVLAKKELDNAQAEIQKITGQVTTAQLEVAGLTNIKTQTEYLTNTID

                      310       320       330       340       350       360
                        |         |         |         |         |         |
MHI241       TAITALQNISNQWYTMGSKYNSLLQNVDSISPNDLVFIKEDLNIAKDSWKNIKDYAEKIY
MHI3178      TAITALQNISNQWYTMGSKYNSLLQNVDSISPNDLVFIKEDLNIAKDSWKNIKDYAEKIY
MHI1440      TAITALQNISNQWYTMGSKYNSLLQNVDSISPNDLVFIKEDLNIAKDSWKNIKDYAEKIY
MHI1541      TAITALQNISNQWYTMGSKYNSLLQNVDSISPNDLVFIKEDLNIAKDSWKNIKDYAEKIY
MHI1668      TAITALQNISNQWYTMGSKYNSLLQNVDSISPNDLVFIKEDLNIAKDSWKNIKDYAEKIY
MHI2970      TAITALQNISNQWYTMGSKYNSLLQNVDSISPNDLVFIKEDLNIAKDSWKNIKDYAEKIY
MHI3173      TAITALQNISNQWYTMGSKYNSLLQNVDSISPNDLVFIKEDLNIAKDSWKNIKDYAEKIY
MHI3225      TAITALQNISNQWYTMGSKYNSLLQNVDSISPNDLVFIKEDLNIAKDSWKNIKDYAEKIY
MHI1430      TAITALQNISNQWYTMGSKYNSLLQNVDSISPNDLVFIKEDLNIAKDSWKNIKDYAEKIY
MHI1444      TAITALQNISNQWYTMGSKYNSLLQNVDSISPNDLVFIKEDLNIAKDSWKNIKDYAEKIY
MHI1758      TAITALQNISNQWYTMGSKYNSLLQNVDSISPNDLVFIKEDLNIAKDSWKNIKDYAEKIY
N5xxxx11     TAITALQNISNQWYTMGSKYNSLLQNVDSISPNDLVFIKEDLNIAKDSWKNIKDYAEKIY

                      370
                        |
MHI241       AEDIKVVDTKKA
MHI3178      AEDIKVVDTKKA
MHI1440      AEDIKVVDTKKA
MHI1541      AEDIKVVDTKKA
MHI1668      AEDIKVVDTKKA
MHI2970      AEDIKVVDTKKA
MHI3173      AEDIKVVDTKKA
MHI3225      AEDIKVVDTKKA
MHI1430      AEDIKVVDTKKA
MHI1444      AEDIKVVDTKKA
MHI1758      AEDIKVVDTKKA
N5xxxx11     AEDIKVVDTKKA
```

Figure S1. Complete sequence alignment of all strains under study.
